# Ethnic Differences in Maternal Adipokines during Normal Pregnancy

**DOI:** 10.3390/ijerph13010008

**Published:** 2015-12-22

**Authors:** Xinhua Chen, Theresa O. Scholl

**Affiliations:** Department of Obstetrics and Gynecology, School of Osteopathic Medicine, Rowan University, Two Medical Center Drive, Suite 390, Stratford, NJ 08084, USA; schollto@rowan.edu

**Keywords:** ethnic difference, adipokine, adiponectin, resistin, normal pregnancy, anthropometric parameters, glucose homeostasis, blood pressure

## Abstract

Two adipokines (adiponectin and resistin) have opposite relations with insulin resistance and inflammation. Our major focus was to determine whether there were detectable ethnic differences in maternal adipokines during pregnancy. We also explored the correlation of the adipokines with maternal glucose homeostasis, blood pressure and anthropometric parameters. Pregnant women (*n* = 1634) were from a large prospective cohort study in Camden NJ (African-American 36.8%; Hispanic 47.6%; Caucasian 15.6%). Serum adiponectin and resistin were measured at entry (week 16.8) and the 3rd trimester (week 30.7) using the Luminex xMapTechnology. Significant differences were observed among ethnic groups, controlling for confounding variables. African American women were exceptional in that they had decreased adiponectin and increased resistin throughout the course of pregnancy (*p* < 0.05 to *p* < 0.0001) and a greater than two fold risk of simultaneously exhibiting low adiponectin (lowest tertile) and high resistin (highest tertile) compared to Caucasians and/or Hispanics. The cohort as a whole and each ethnic group showed similar negative correlations between adiponectin, and glucose homeostasis, blood pressure and anthropometric parameters but there was lesser correspondence with resistin. Our data underscore the need for further research on ethnic variation in adipokines and other physiologic biomarkers during complicated and uncomplicated pregnancy.

## 1. Introduction

Adiponectin and resistin are hormones that are produced and secreted exclusively by the adipocyte [1,2]. The two hormones have opposite relations with insulin resistance—a mechanism that underlies many conditions including the development of cardiovascular disease (CVD) [3,4,5]. Plasma adiponectin is a strong anti-inflammatory marker whereas plasma resistin is positively related to inflammation and insulin resistance [2,3,4,6].

While ethnic differences in the body composition, insulin resistance and metabolic syndrome are well known [7,8,9]; the fundamental reason for these differences is not. It is also unclear if dissimilarities in glucose, lipids and inflammation contribute to the ethnic disparity in chronic disease [10,11,12,13]. While some studies suggest that adiponectin and resistin may be involved in serious complications of pregnancy where disparities also exist—such as gestational diabetes mellitus (GDM) and preeclampsia [14,15,16,17], underlying ethnic variability has not been described in pregnant women. Thus, the purpose of this study was to prospectively investigate ethnic variation in maternal adiponectin and resistin levels during normal pregnancy. We used data from a large cohort that included pregnant Hispanic, African American and Caucasian women. We also examined, by ethnicity, the associations of the two adipokines with maternal anthropometry, blood pressure and glucose homeostasis during early and late gestation.

## 2. Materials and Methods

### 2.1. Study Design and Ethics Statement

We conducted a prospective epidemiological study of young, generally healthy pregnant women residing in one of the poorest cities in the continental United States [18]. The cohort of study participants enrolled between 1996 and 2006 was recruited from among patients enrolling at the Osborn Family Health Center, Our Lady of Lourdes Medical Center and St John the Baptist prenatal clinic in Camden, NJ. The institutional review board at the University of Medicine and Dentistry of New Jersey (which later became Rowan University School of Osteopathic Medicine in 2013) approved the study protocol. Informed written consent was obtained from each participant at enrollment (on average at gestational week 13.48 ± 5.18, mean ± SD) after explanation of the nature and purpose of the study.

### 2.2. Subjects

A total of 3.5% of the women who were screened had serious non-obstetric problems (e.g., lupus, type 1 or 2 diabetes, seizure disorders, malignancies, acute or chronic liver disease, drug or alcohol abuse and psychiatric problems) were not eligible. Eighty percent of the patients who were eligible agreed to participate. A total of 8.3% of participants dropped out after enrollment due either to a move from the area or to an early pregnancy loss. A final total of 1634 participants with specimens collected at entry and the 3rd trimester were included to this analysis.

### 2.3. Data and Blood Sample Collection

Participants were scheduled to see study research assistants before or after their regular prenatal visits at entry, gestational weeks 20 and 28. Data on socioeconomic, demographic and lifestyle were obtained by interview at entry to care and updated at gestational weeks 20 and 28. Interview data, dietary data and anthropometrics were collected in person by research assistants at these three times.

Ethnicity was self-defined. Blood pressure was measured by clinic staff at each visit and abstracted from the medical records. Body mass index (BMI) (kg/m^2^) was computed based on self-reported pregravid weight; height was measured at entry to prenatal care. Maternal obesity was defined as BMI ≥ 30. Skinfold thickness was measured by a skinfold caliper (Cambridge Scientific Industries, Inc., Cambridge, MA) and upper arm fat was computed with measures of arm circumference and triceps thickness as described [19]. Three different points of the skinfold thickness including triceps, subscapular and suprailiac were totaled.

Fasting blood samples (>8 h) were drawn at entry (gestational weeks 16.85 ± 5.46, mean ± SD) and during the 3rd trimester (gestational weeks 30.74 ± 3.58). A standardized protocol for biological specimen collection is used for the Camden Study which includes the serum preparation used in the current study. The serum was immediately transferred into clean polypropylene cryovials and stored at –80 °C. The serum samples were aliquoted and not thawed until assayed.

### 2.4. Analytic Procedures

#### 2.4.1. Adipokine Assay

Adiponectin and resistin concentrations were analyzed on the Magpix system using the Luminex xMAP technology (Luminex Corporation, Austin, TX, USA). Serum concentrations of adiponectin and resistin were determined simultaneously by the human adipokine 2-plex magnetic bead panel from EMD Millipore Corporation (HADK1MAG-61K-2, Billerica, MA, USA). According to the manufacturer, there was no or negligible cross-reactivity between the antibodies for an analyte and any of the other analytes in this panel. The minimal detectable concentrations were 11 pg/mL for adiponectin and 2.2 pg/mL for resistin. The coefficient variations within and between assays was 4.4% and 10.1% for adiponectin and 5.5% and 11.3% for resistin. All analyses were performed according to the manufactures’ protocols and done in duplicate. The concentrations were calculated from best-fit standard curves generated from calibrators for each analyte in each assay.

#### 2.4.2. Glucose Homeostasis Measures

Fasting plasma glucose was measured with the glucose oxidase method (glucose reagent from Sigma Diagnostics, St. Louis, MO, USA) on a spectrophotometer at a wavelength of 505 nm. Serum insulin was determined by radioimmunoassay (RIA) using a kit with a specific antibody that crossreacts only minimally (<0.2%) with proinsulin and has a high sensitivity (2 μμU/mL or 12 pmol/L). Plasma *C*-peptide was determined by an RIA kit with high sensitivity (0.1 ng/ml or 0.033 nmol/l) and low crossactivity to proinsulin (<4%) (Linco, St. Charles, MO, USA). The coefficient of variation within and between assays was 3.2% and 6.3% for *C*-peptide, 3.5% and 6.5% for insulin, 1.5% and 3.0% for glucose. The homeostasis model of assessment for insulin resistance (HOMA-IR) was computed from the same day’s fasting specimens of glucose and insulin divided by a constant (22.5), under the assumption that young normal subjects have an insulin resistance of 1.0 [20,21,22].

### 2.5. Statistical Analysis

Maternal characteristics among ethnic groups were compared by ANOVA (for continuous variables) and *x*^2^ tests (for categorical variables). ANOVA was used to assess the significance of linear trend and compare mean levels of each adipokine among ethnic groups after controlling for potential confounding variables. The Bonferroni procedure was used to correct for multiple comparisons. We divided each adipokine into tertiles and used multiple logistic regression analysis to estimate the Odds Ratio (OR) and 95% confidence interval (CIs) for the likelihood of decreased adiponectin (the lowest tertile) and increased resistin (the highest tertile) with ethnicity. Separate models were performed to examine differences in adipokine levels at entry to care and in the 3rd trimester among ethnic groups.

The relationship between adipokine levels with glucose homeostasis as well as anthropometric parameters was assessed by computing Pearson’s *r* for all women as well as for each ethnic group. The relation for these same parameters with low adiponectin (lowest tertile) combined with high resistin (highest tertile) was assessed by a point biserial correlation (*r_pb_*). Multivariable models were controlled for potential confounding variables including maternal BMI, age, parity, and cigarette smoking. Potential confounders were defined as those which altered the adjusted odds ratio or regression coefficient by at least 10%, and assessed by comparing crude and adjusted coefficients.

The statistical significant level was defined as *p* < 0.05. All statistical analyses were performed using SAS v.9.2 (SAS Institute, Inc., Cary, NC, USA).

## 3. Results and Discussion

Selected characteristics of study participants are shown in Table 1. African-American women were younger and had higher pre-pregnant BMI comparing to Caucasian or Hispanic women, and Caucasians were about twice as likely to smoke as other gravidas. Other variables including obesity, the sum of skinfolds, and blood pressure were comparable among African American, Hispanic and Caucasian women.

**Table 1:** Characteristics of study participants at entry by ethnicity ^a^.

Characteristics	All Subjects	African American (*n* = 602)	Hispanic (*n* = 777)	Caucasian (*n* = 255)
Age (year.) ^b^	22.03 ± 5.29	21.38 ± 5.02	22.19 ± 5.27	23.11 ± 5.75
Pre-pregnant BMI (kg/m^2^) ^c^	25.73 ± 6.30	26.15 ± 7.01	25.74 ± 5.88	24.72 ± 5.65
Obese (BMI ≥ 30)	329 (20.13)	135 (22.43)	148 (19.05)	46 (18.04)
Sum of skinfolds (mm)	70.62 ± 29.56	72.19 ± 32.45	70.33 ± 28.05	67.95 ± 26.69
Upper arm fat area (mm)	28.40 ± 16.24	29.74 ± 18.75	27.59 ± 14.80	27.69 ± 13.79
SBP (mmHg)	112.20 ± 11.35	113.81 ± 11.65	110.78 ± 10.90	112.74 ± 11.49
DBP (mmHg)	70.21 ± 8.66	71.21 ± 9.07	69.56 ± 8.18	69.84 ± 8.91
Nulliparous	640 (39.17)	225 (37.38)	311 (40.03)	104 (40.78)
Cigarette smoking ^b^	310 (18.97)	117 (19.44)	95 (12.23)	98 (38.43)

SBP, systolic blood pressure; DBP, diastolic blood pressure; ^a^ Data are mean ± SD or *n* (%). Twelve Asian women are included in the Caucasian group; ^b^ *p* for trend <0.0001; ^c^ *p* for trend <0.01.

### 3.1. Comparison of Adipokines among Ethnic Groups

Adiponectin concentration decreased between entry and the 3rd trimester in all women from the cohort as well as for each ethnic group (*p* < 0.0001 for each) whereas resistin concentrations remained unchanged (*p* > 0.05 for each).

Significant differences in serum concentrations of adiponectin and resistin were observed among ethnic groups after controlling for several confounding variables including pre-pregnant BMI (Table 2). A graded pattern of adiponectin was obtained where African American women had the lowest adiponectin level compared to Hispanic and/or Caucasians both at entry and during the 3rd trimester. The difference between Hispanic and Caucasian women was also significant (*p* < 0.05) with Hispanics having lower concentrations.

**Table 2:** Difference in adiponectin and resistin concentrations by ethnicity ^a^.

Ethnic Group	Adiponectin (µg/mL)		Resistin (ng/mL)	
	Entry	3rd Trimester	Entry	3rd Trimester
All subjects	17.66 ± 0.21	14.75 ± 0.20	47.49 ± 0.63	48.22 ± 0.62
African American	16.49 ± 0.36 ^b,c^	13.23 ± 0.29 ^b,e^	50.32 ± 1.10 ^e^	50.35 ± 1.09 ^c^
Hispanic	17.90 ± 0.32 ^d^	15.16 ± 0.33 ^d^	45.28 ±1.11 ^d^	46.61 ± 0.96
Caucasian	19.34 ± 0.57	16.72 ± 0.52	49.43 ± 1.73	49.24 ± 1.72

^a^ Data are mean ± SE. Models were adjusted for maternal age, pre-pregnancy BMI, parity and cigarette smoking; ^b^ *p* < 0.0001 *vs.* Caucasian; ^c^ *p* < 0.01 *vs.* Hispanic; ^d^ *p* < 0.05 *vs.* Caucasian; ^e^ *p* < 0.0001 *vs.* Hispanic.

A different pattern was shown for resistin. Hispanics had the lowest resistin level and African American women had the highest (*p* < 0.0001 at entry and *p* < 0.01 at 3rd trimester *vs.* Hispanics). While levels in African Americans did not differ significantly from levels in Caucasians, entry levels in Hispanics were significantly lower than Caucasians.

### 3.2. Association of Ethnicity with Low Adiponectin (Lowest Tertile) and High Resistin (Highest Tertile)

After control for potential confounding variables and using Caucasians as reference (Table 3), African American women were twice as likely to have low adiponectin (lowest tertile) at entry and in the 3rd trimester. The results did not change significantly after controlling for pre-pregnant BMI (model 2). Although Hispanics had lower adiponectin than Caucasians (Table 2), their likelihood of being in the lowest tertile was not significant except during the 3rd trimester and only when BMI was not controlled (adjusted odds ratio (AOR) 1.43, 95% confidence interval (CI) 1.02, 2.00, Table 3, model 1).

The results were not statistically significant when testing for ethnic differences in increased resistin (highest tertile) using the Caucasians as the reference group. Because the distribution of resistin among ethnic groups was different from adiponectin, in that Hispanics had the lowest resistin level, we analyzed data by using Hispanics as reference (Table 4). African American women were more likely to be in the highest resistin tertile compared to Hispanics both at entry and in the 3rd trimester when models were fully controlled (AOR 1.47, 95% CI 1.17, 1.85 for entry; AOR 1.29, 95% CI 1.03, 1.62 for 3rd trimester, Table 4, model 2). The adjusted odds ratios were not significant comparing Caucasians to Hispanics.

**Table 3:** Multiple logistic regression analysis for the associations of decreased adiponectin with ethnicity.

Low Adiponectin ^a^	Unadjusted *n* (%)	AOR (95% CI) Model 1 ^b^	AOR (95% CI) Model 2 ^c^
Entry			
African American	248 (41.2)	2.07 (1.48, 2.89)	1.86 (1.32, 2.63)
Hispanic	235 (30.24)	1.23 (0.90, 1.74)	1.15 (0.82, 1.61)
Caucasian	66 (25.88)	Reference	Reference
3rd Trimester			
African American	258 (42.86)	2.61 (1.85, 3.66)	2.41 (1.70, 3.41)
Hispanic	230 (29.60)	1.43 (1.02, 2.00)	1.34 (0.95, 1.89)
Caucasian	59 (23.14)	Reference	Reference

AOR, adjusted odds ratio; 95% CI, 95% confidence interval, same in Table 4 and Table 5; **^a^** Low adiponectin was defined as the lowest tertile (≤12.85 µg/mL at entry and ≤10.35 µg/mL at 3rd trimester) as compared to tertiles one and two pooled; **^b^** Model 1: Adjusted for age, parity and cigarette smoking; **^c^** Model 2: Additional adjustment for pre-pregnant BMI.

**Table 4:** Multiple logistic regression analysis for the associations of elevated resistin with ethnicity.

High Resistin ^a^	Unadjusted *n* (%)	AOR (95% CI) Model 1 ^b^	AOR (95% CI) Model 2 ^c^
Entry			
African American	227 (37.71)	1.49 (1.18, 1.87)	1.47 (1.17, 1.85)
Hispanic	228 (29.34)	Reference	Reference
Caucasian	91 (35.64)	1.18 (0.87, 1.61)	1.21 (0.89, 1.65)
3rd Trimester			
African American	219 (36.38)	1.31 (1.04, 1.65)	1.29 (1.03, 1.62)
Hispanic	233 (29.99)	Reference	Reference
Caucasian	94 (36.86)	1.24 (0.91, 1.69)	1.28 (0.94, 1.74)

**^a^** High resistin was defined as the highest tertile (≥52 ng/mL at entry and ≥53 ng/mL at 3rd trimester) compared to tertiles one and two pooled; **^b^** Models were adjusted for age, parity and cigarette smoking; **^c^** Additional adjustment for pre-pregnant BMI.

Seventy three women developed GDM during late pregnancy (*n* = 12, 42, 19 for African American, Hispanics and Caucasians, respectively). After excluding women who developed GDM during the 3rd trimester, these results again remained unchanged. For example, the AOR was 2.64 for model 1 (95% CI 1.82, 3.84) and was 2.43 for model 2 (95% CI 1.67, 3.55) in African Americans compared to Caucasians (Table 3).

In addition, African Americans appeared to have a unique difference in that they had an increased risk of simultaneously having both low adiponectin and high resistin throughout the course of pregnancy compared to Caucasians (AOR 2.23, 95% CI 1.28, 3.87 at entry and AOR 1.70, 95% CI 1.01, 2.89 for the 3rd trimester, Table 5, model 2) as well as compared to Hispanics (AOR 2.19, 95% CI 1.52, 3.17 at entry and AOR 1.83, 95% CI 1.27, 2.63, Table 6, model 2).

**Table 5:** Association of combination of low adiponectin and high resistin with ethnicity ^a^.

Ethnic Group	Unadjusted	AOR (95% CI)	
Entry	*n* (%)	Model 1 ^b^	Model 2 ^c^
African American	87 (14.45)	2.55 (1.48, 4.39)	2.23 (1.28, 3.87)
Hispanic	55 (7.08)	1.09 (0.62, 1.92)	1.02 (0.57, 1.80)
Caucasian	18 (7.06)	Reference	Reference
3rd trimester			
African American	80 (13.29)	1.89 (1.12, 3.19)	1.70 (1.01, 2.89)
Hispanic	58 (7.46)	1.00 (0.58, 1.72)	0.93 (0.54, 1.61)
Caucasian	20 (7.84)	Reference	Reference

^a^ The lowest tertile of adiponectin (≤12.85 µg/mL for entry and ≤10.35 µg/mL for 3rd trimester) and the highest tertile of resistin (≥52 ng/mL for entry and ≥53 ng/mL for 3rd trimester) were combined *vs.* other tertiles, using Caucasians as reference; ^b^ Model 1: Adjusted for age, parity and cigarette smoking; ^c^ Model 2: Additionally adjustment for pre-pregnant BMI.

**Table 6:** Association of combination of low adiponectin and high resistin with ethnicity ^a^.

Ethnic Group	Unadjusted	AOR (95% CI)	
Entry	*n* (%)	Model 1 ^b^	Model 2 ^c^
African American	87 (14.45)	2.34 (1.63, 3.36)	2.19 (1.52, 3.17)
Hispanic	55 (7.08)	Reference	Reference
Caucasian	18 (7.06)	0.92 (0.52, 1.62)	0.98 (0.56, 1.74)
3rd trimester			
African American	80 (13.29)	1.89 (1.31, 2.71)	1.83 (1.27, 2.63)
Hispanic	58 (7.46)	Reference	Reference
Caucasian	20 (7.84)	1.00 (0.58, 1.72)	1.07 (0.62, 1.86)

^a^ The lowest tertile of adiponectin (≤12.85 µg/mL for entry and ≤10.35 µg/mL for 3rd trimester) and the highest tertile of resistin (≥52 ng/mL for entry and ≥53 ng/mL for 3rd trimester) were combined *vs.* other tertiles, using Hispanics as reference; ^b^ Model 1: Adjusted for age, parity and cigarette smoking; ^c^ Model 2: Additionally adjustm ent for pre-pregnant BMI.

### 3.3. The Correlation between Adipokine Levels with Maternal Factors

**Figure 1:**
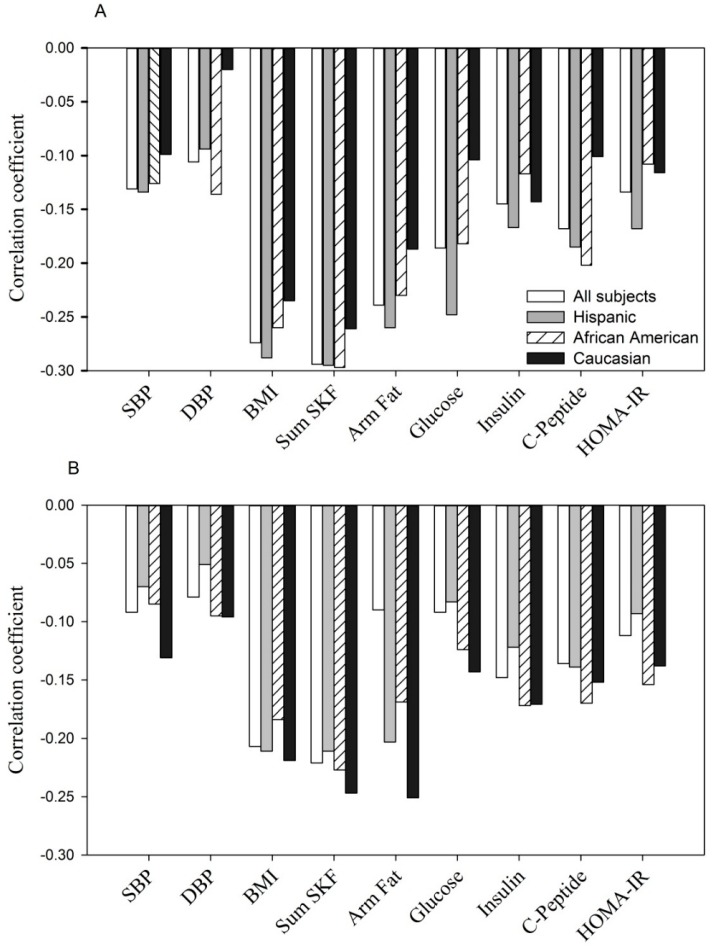
(**A**, at entry; **B**, during the 3rd trimester). The relationship between serum adiponectin (µg/mL) with anthropometric and glycemic parameters.

Finally, adiponectin concentrations were significantly and negatively correlated with maternal BMI, sum of skinfolds, arm fat area, systolic and diastolic blood pressure and, glucose homeostasis parameters (fasting glucose, insulin, *C*-peptide and HOMA-IR) at entry (Figure 1A, *p* < 0.05 to *p* < 0.0001 for *r* = −0.11 to *r* = −0.30) and during the 3rd trimester (Figure 1B, *p* < 0.05 to *p* < 0.0001, *r* = −0.10 to *r* = −0.27). A similar relationship was observed for each of the ethnic groups.

Shown are Pearson correlation coefficients in all women and by ethnicity. For fasting glucose, insulin and *C*-peptide (*n* = 1323).

**Figure 2:**
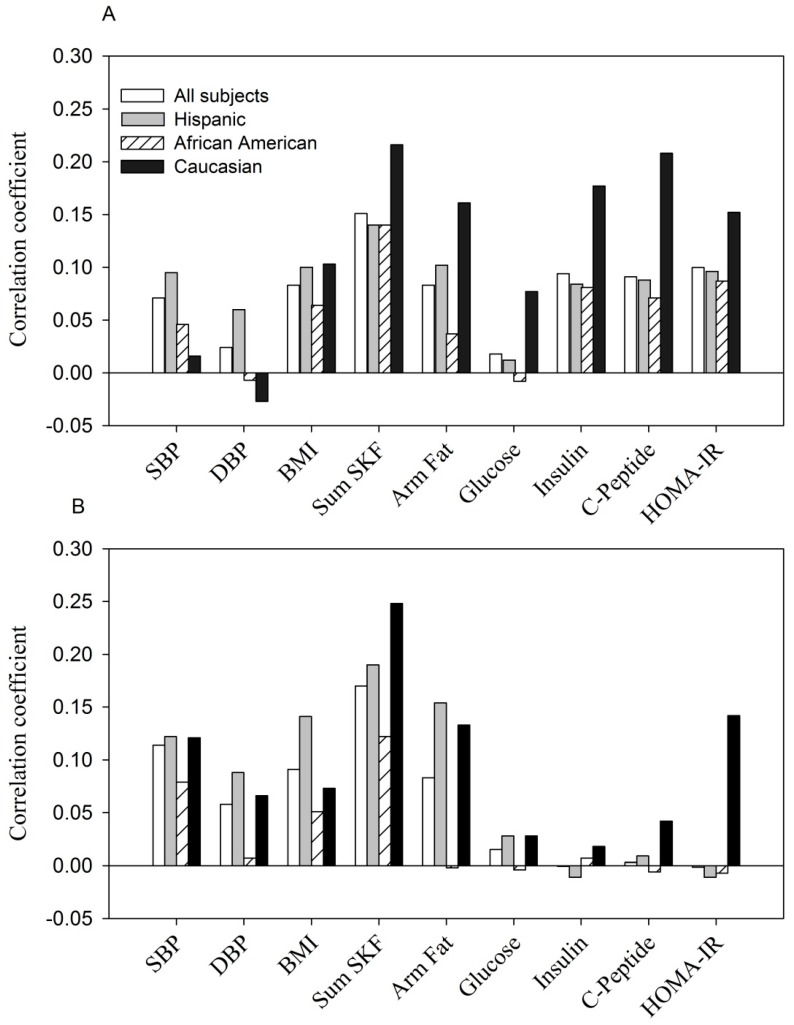
(**A**, at entry; **B**, during the 3rd trimester). The relationship between serum resistin (ng/mL) with anthropometric and glycemic parameters.

**Figure 3:**
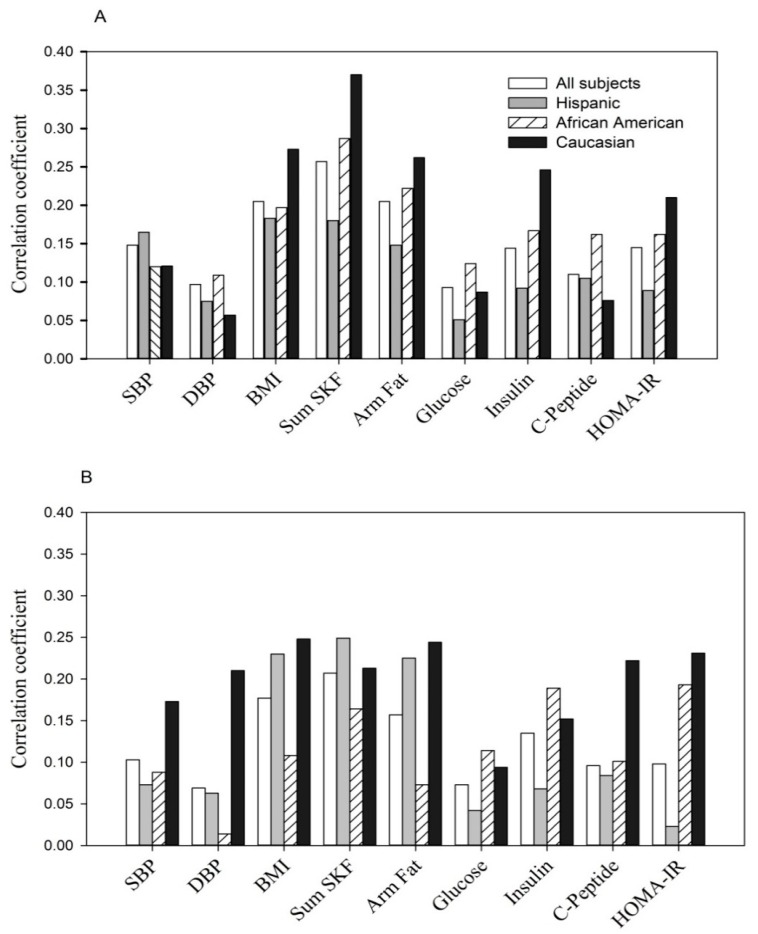
(**A**, at entry; **B**, during the 3rd trimester). The relationship of variable combined low adiponectin (lowest tertile) with high resistin (highest tertile) to anthropometric and glycemic parameters.

SBP, systolic blood pressure; DBP, diastolic blood pressure; Sum SKF, sum of skinfolds; Arm fat, upper arm fat area; HOMA-IR, the homeostatic model assessment of insulin resistance (same as in Figure 2 and Figure 3).

- Negative correlation between adiponectin concentration with selected parameters at entry: *p* < 0.05 to *p* < 0.0001 for each of parameters tested in all women, Hispanics and African Americans. In Caucasians, *p* < 0.05 to *p* < 0.001 with BMI, sum of skinfolds, arm fat area and insulin; but *p* > 0.05 for SBP, DBP, *C*-peptide, glucose and HOMA-IR.
- Negative correlation between adiponectin concentration with selected parameters during the 3rd trimester: *p* < 0.05 to *p* < 0.0001 for each of parameters tested in all women, Hispanics, African Americans and Caucasians, except for DBP, fasting glucose in Hispanics (*p* > 0.05) and DBP, glucose and HOMA-IR in Caucasians (*p* > 0.05).

In contrast, the relationship between resistin and the same parameters was weaker and less consistent (Figure 2). At entry, in all subjects, fasting glucose and diastolic blood pressure were not significantly correlated with resistin while SBP, BMI, sum of skinfolds, arm fat area, fasting *C*-peptide, insulin and HOMA-IR were modestly related (*p* < 0.05 to *p* < 0.0001, *r* = +0.07 to *r* = +0.15 (Figure 2A). The pattern of correlation was similar but much weaker when examined by ethnicity. During the 3rd trimester, significant correlations were obtained between resistin and systolic and diastolic blood pressure, sum of skinfolds, arm fat area and BMI in all women (*p* < 0.05 to *p* < 0.001, *r* = +0.08 to *r* = +0.20). A similar pattern was observed in Hispanics and Caucasians. In African American women, resistin was correlated only with the sum of skinfolds (*r* = +0.12, *p* < 0.01). None of the glucose homeostasis parameters reached statistical significance overall or by ethnic groups (Figure 2B).

Shown are Pearson correlation coefficients in all women and by ethnicity.

- The positive correlation between resistin concentration with selected parameters at entry: *p* < 0.05 to *p* < 0.0001 for each parameter tested in all women, Hispanics and Caucasians, except for DBP, fasting glucose in all women and Hispanics (*p* > 0.05); for SBP, DBP, BMI and fasting glucose in Caucasians (*p* > 0.05). The correlations were all non-significant in African Americans except for sum of skinfolds (*p* < 0.01).
- The positive correlation between resistin concentration with selected parameters at the 3rd trimester: *p* < 0.05 to *p* < 0.0001 for SBP, DBP, BMI, sum of skinfolds and arm fat area in all women and Hispanics; *p* < 0.01 for sum of skinfolds in African Americans; *p* < 0.001 and *p* < 0.05 for sum of skinfolds and arm fat area in Caucasians. Other parameters were all non-significant.

The correlation between the same parameters with the combination of low adiponectin and high resistin (Figure 3) was consistently positive and observed in all women (*p* < 0.05 to *p* < 0.0001, *r_pb_* = +0.07 to *r_pb_* = +0.37) as well as by maternal ethnicity.

Shown are point biserial correlation (*r_pb_*) coefficients in all women and by ethnicity.

- The positive correlation at entry: *p* < 0.05 to *p* < 0.0001 for each of parameters except fasting glucose in Hispanics (*p* > 0.05) and DBP, fasting glucose and *C*-peptide in Caucasians (*p* > 0.05).
- The positive correlation at the 3rd trimester: *p* < 0.05 to *p* < 0.0001 for each of parameters with the exception of DBP, fasting glucose and insulin in Hispanics (*p* > 0.05), DBP and arm fat area in African Americans (*p* > 0.05) and fasting glucose in Caucasians (*p* > 0.05).

### 3.4. Discussion

Our study is unique in that we examined the relation of ethnic variation in two adipokines with multiple parameters of maternal glucose homeostasis, blood pressure and with several anthropometric measures taken in early and later pregnancy. We used data from a large prospective cohort of 1634 normal pregnant women. We found that African American women had decreased adiponectin and increased resistin levels during early and late gestation. Hispanics also had decreased adiponectin, but their resistin levels were lower than African Americans (at both time points) and Caucasians (at entry alone). Thus, African Americans were found to have a higher prevalence of an exceptional pattern of adipokines that coupled low adiponectin with high resistin throughout the course of pregnancy.

While ethnic differences in adiponectin have infrequently been reported during pregnancy they have been described in non-pregnant subjects. Smith *et al.* found that healthy African American women had lower adiponectin (*p* < 0.01) accompanied by reduced expression of several genes involved in adipose tissue adipogenesis and lipogenesis when compared to Caucasians [23]. Lower adiponectin concentrations have also been reported in South Asians and Aboriginal Canadians compared to Europeans or Caucasians [24,25]. Findings from one of these studies (albeit in males and non-pregnant females) of low adiponectin with increased insulin resistance is similar to what is found in the pregnant state [25].

In an earlier case-control study, we described lower adiponectin among pregnant African Americans regardless of whether they developed GDM, had hyperglycemia without overt GDM or were normal controls; the difference was especially detectable during 3rd trimester [14]. In this larger prospective cohort, we confirmed that African American women had significantly decreased mean levels of adiponectin (*versus* Hispanics or Caucasians) and a two-fold increase of being in the lowest tertile (*vs.* Caucasians) (Table 2 and Table 3). These results were independent of several potential confounding variables including BMI. To our knowledge, the current study is the first large scale and prospective demonstration of ethnic differences in serum adiponectin for US pregnant women.

Likewise data describing ethnic differences in resistin during pregnancy are uncommon. The only report of which we are aware showed no ethnic differences in resistin and other inflammatory cytokines including TNF-α and *C*-reactive protein in non-pregnant Aboriginals compared to white Canadians [24].

Among postmenopausal women and other subjects, elevated serum resistin is associated with increased risk of type 2 diabetes, metabolic syndrome and ischemic stroke [3,4,5,6], but results from pregnancy are conflicting. Small scale studies conducted late in gestation, usually following a diagnosis of gestational diabetes, found resistin to be increased, decreased or unchanged compared to controls [26,27,28]. As far as we are aware, no other investigators have reported the pattern we detected among African Americans: An increased prevalence in the simultaneous combination of low adiponectin with high resistin.

In the current study, adiponectin was significantly and negatively correlated at both time points with all maternal factors examined including BMI, skinfolds, systolic and diastolic blood pressure, fasting levels of glucose, insulin, *C*-peptide and HOMA-IR (Figure 1). When examined by ethnicity, each group showed a similar correspondence between maternal factors and adiponectin.

Apart from positive correlations between resistin and maternal adiposity the association between resistin and the same maternal factors was weak and inconsistent (Figure 2). No significant correlation was observed between resistin and glucose homeostasis variables during the 3rd trimester for any of the three ethnic groups. Likewise, the Hyperglycemia and Adverse Pregnancy Outcome study showed that resistin was positively related to maternal BMI but not with fasting or 1 or 2 h glucose [29]. Thus, maternal resistin concentration in non-GDM pregnancy may reflect adipose or fat mass. The role of resistin in normal pregnancy, a condition with dramatic changes in metabolic demand and cardiovascular function, remains poorly defined when compared to the non-pregnant state.

Several studies, none from pregnant women, have shown that ethnic differences in adiponectin parallel ethnic variation in risk factors for CVD such as visceral fat distribution and insulin resistance [10,24,25]. Since adiponectin is protective against inflammation and anthogenesis [1,5,7], we hypothesize that decreased adiponectin in African American pregnant women may prognosticate reduced protection against development of serious complications such as GDM. 

Inflammatory mediators including endothelial adhesion molecules have been associated with adverse pregnancy outcomes like preterm delivery [15,30,31]. More research is needed to confirm the extent to which ethnic differences in adipokines and in their pattern contribute to adverse outcomes. Likewise it would be important to examine links between maternal life style and diet to ethnic variation in the adipokines, and to ethnic disparities during pregnancy.

## 4. Conclusions

In summary, we found ethnic differences in adiponectin and resistin in normal pregnant women. African American women, in particular, had a higher prevalence of simultaneously decreased adiponectin and increased resistin throughout pregnancy. Maternal resistin displayed a different pattern from adiponectin in relation to anthropometry, blood pressure and glucose homeostasis. Our data underscore the need for further research linking ethnic variation in adipokines and other physiologic biomarkers with ethnic disparities during pregnancy.
